# Early Emergence and Dispersal of Delta SARS-CoV-2 Lineage AY.99.2 in Brazil

**DOI:** 10.3389/fmed.2022.930380

**Published:** 2022-06-17

**Authors:** Camila Malta Romano, Cristina Mendes de Oliveira, Luciane Sussuchi da Silva, José Eduardo Levi

**Affiliations:** ^1^Hospital das Clinicas da Faculdade de Medicina da Universidade de São Paulo (HCFMUSP), São Paulo, Brazil; ^2^Instituto de Medicina Tropical de São Paulo, Moléstias Infecciosas, Universidade de São Paulo, São Paulo, Brazil; ^3^Research & Development, Dasa Laboratories, Dasa, Brazil

**Keywords:** SARS-CoV-2, Delta, variants of concern, sub-lineages, dispersal

## Abstract

The year of 2021 was marked by the emergence and dispersal of a number of SARS-CoV-2 lineages, resulting in the “third wave” of COVID-19 in several countries despite the level of vaccine coverage. Soon after the first confirmed cases of COVID-19 by the Delta variant in Brazil, at least seven Delta sub-lineages emerged, including the globally spread AY.101 and AY.99.2. In this study we performed a detailed analysis of the COVID-19 scenario in Brazil from April to December 2021 by using data collected by the largest private medical diagnostic company in Latin America (Dasa), and SARS-CoV-2 genomic sequences generated by its SARS-CoV-2 genomic surveillance project (GENOV). For phylogenetic and Bayesian analysis, SARS-CoV-2 genomes available at GISAID public database were also retrieved. We confirmed that the Brazilian AY.99.2 and AY.101 were the most prevalent lineages during this period, overpassing the Gamma variant in July/August. We also estimated that AY.99.2 likely emerged a few weeks after the entry of the B.1.617.2 in the country, at some point between late April and May and rapidly spread to other countries. Despite no increased fitness described for the AY.99.2 lineage, a rapid shift in the composition of Delta SARS-CoV-2 lineages prevalence in Brazil took place. Understanding the reasons leading the AY.99.2 to become the dominant lineage in the country is important to understand the process of lineage competitions that may inform future control measures.

## Introduction

Coronavirus disease 2019 (COVID-19) emerged in late 2019. It is a life threatening viral respiratory infection caused by a novel betacoronavirus (SARS-CoV-2) of probable bat origin, which is related to the virus responsible for the Severe Acute Respiratory Syndrome (SARS) outbreak in 2002/2003 in humans (1, 2). By March 15th, 2022 more than 456 million confirmed cases and about 6 million deaths were reported by the World Health Organization (WHO) (3).

Soon after its emergence, the virus caused a huge global wave with a high death rate mainly among the elderly. The partial immunologic protection against reinfection and rapid viral evolution allowed for subsequent waves of higher incidence and mortality led by the variants of concern (VOC) Alpha, Beta and Gamma, that emerged almost simultaneously in the United Kingdom, South Africa and the Brazilian Amazon, respectively (4).

In October 2020 the first case of COVID-19 caused by the Delta variant (B.1.617.2) was identified in India and this lineage was further designated as a new VOC by WHO (4). In June 2021 this variant was already detected in 96 countries including Brazil that confirmed the first case in late April 2021. At that time, the Gamma variant that emerged in December 2020 in the Amazon state was still predominant in Brazil (5, 6). Today, more than 240 B.1.617.2 sub-lineages are described (cov-lineages/pango) as a result of collaborative genomic surveillance programs.

Dasa is the largest medical diagnostic company in South America, having performed since February 2020, more than 5.2 million COVID-19 RT-PCRs on samples from all over Brazil. In 2021, Dasa implemented a genomic surveillance project entitled GENOV (https://dasa.com.br/en/genov/) that already counts more than 10 thousand SARS-CoV-2 complete genome sequences public available at GISAID (gisaid.org/epicov).

Brazil is among the countries most affected by the COVID-19 pandemic, with 660 thousand deaths, second only behind the USA, which accumulated ~1 million deaths until March 2022.

Previous estimates suggested at least three introductions of Delta in Brazil, all of them around April 2021 (7). Since then, seven sublineages that have likely originated in Brazil (AY.34.1.1; AY.43.1;.2 and.3; AY.46.3; AY.99.1 and.2 and AY.101) were described. Among these lineages, the AY.99.2 was the most dominant one during the Delta wave in the country, reaching 58% of all Delta sublineages sampled during the period (8). For instance, AY.101, the second more prevalent Brazilian Delta sublineage, reached only 7.6% in the same period. The states of Paraíba, Rio de Janeiro, and Distrito Federal presented the highest prevalence of AY.99.2 sublineage, with 90, 79, and 83% respectively (8).

According to the GISAID public database the very first AY.99.2 sampled in Brazil dates back to April 2021 (EPI_ISL_8057837), though we could not confirm the accuracy of the sampling date with the submitters. Nevertheless, by May 2021 AY.99.2 was sampled in three different states in Brazil, indicating its spread throughout the country (gisaid.org/epicov).

By using data from DASA and SARS-CoV-2 genomic sequence generated by the SARS-CoV-2 genomic surveillance project from Dasa (GENOV), we describe the COVID-19 scenario in Brazil from April to December 2021 and estimate the time of the origin of AY.99.2.

## Methods

### Scenario

Naso/oropharyngeal swabs were collected between April and December 2021 from subjects seeking one of the 900 Dasa sampling outposts distributed throughout Brazil, for routine SARS-CoV-2 RT-PCR testing. This population presented the full range of the clinical spectrum, from severely ill hospitalized patients to asymptomatic travelers. Swabs were dipped in 3 ml of sterile saline and transported under refrigeration (2–8°C) to the central laboratory located in Barueri, São Paulo state, Brazil. For the GENOV surveillance program, the choice of samples aimed to statistically represent all regions of the country, reflecting the local incidence of SARS-CoV-2 in the period. For technical reasons, only positive samples with Ct <30 (Cycle Threshold) values were selected, corresponding to viral loads that allow the sequencing of the complete genome with acceptable quality.

Sequences were generated using Illumina COVIDSeq Kit (Illumina, CA, USA) and the NovaSeq 6,000 platform (Illumina, CA, USA). Bioinformatic analyses were performed with Illumina^®^ DRAGEN COVID Lineage App (version 3.5.3) in Basespace Sequence Hub. Consensus fasta sequences that passed the DRAGEN COVID Lineage pipeline's quality control were submitted to GISAID and GenBank databases. Supplementary Material contains the summarized information regarding the sampling date of the sequences.

### Sampling Global AY.99.2

Looking for global AY.99.2 in GISAID (uploaded until January 19th 2022), a total of 21K complete genomes (excluding low coverage and incomplete information on the place or sampling date) were found. Of those, 20.7 K were from Brazil. North America and Europe only counted for 289 and 174 AY.99.2 isolates, respectively. South American neighbor countries altogether uploaded 120 AY.99.2 sequences until the date we checked, being mostly from Chile (*n* = 66) and Argentina (*n* = 36).

### Phylogenetic and Coalescent Analysis

A dataset using a subset of the GISAID retrieved sequences (*n* = 400) was built and used to reconstruct a global phylogenetic tree. All countries where at least three isolates from this lineage were detected were represented in the dataset. The sequences were multiple aligned using MAFFT v7.407 (9) and after careful visual inspection, the dataset was submitted to maximum likelihood (ML) phylogenetic analysis with IQ-TREE 2 (10) under the GTR + I + G nucleotide substitution model, selected as the best-fitting one by ModelFinder implemented in IQTREE 2. The branches support values were accessed from the ultrafast bootstrap with 1,000 replicates and the final tree was visualized with FigTreev1.4.4 (http://tree.bio.ed.ac.uk/software/figtree/).

The time of the most recent common ancestor (tMRCA) for the Brazilian lineage AY.99.2 was estimated using a Bayesian MCMC approach implemented in BEAST 1.10.4 (11) for a subset of AY.99.2 Brazilian sequences sampled from May 2021 to January 2022. The earliest AY.99.2 available at GISAID dates back to April 2021. However, as we could not confirm the validity of the sampling date for this sequence, it was not included in the Bayesian analysis.

The Brazilian dataset was mostly but not exclusively built using the genomes generated by the GENOV surveillance project. The Bayesian Skyline (BSL) coalescent method was performed under relaxed uncorrelated exponential molecular clock using time-stamped data scaled in months under GTR + I + G nucleotide substitution model. Since the SARS-CoV-2 substitution rate can vary over time we set the uced.mean with uniform prior (5.0−10E-4), as it covers the range of the substitution rates estimated previously from SARS-COV-2 (12–14). Convergence of parameters was inspected with Tracer v.1.7.2 with uncertainties addressed as 95% highest probability density (HPD) intervals. After 50 million runs, the trees sampled at every 5,000 steps were summarized in a maximum clade credibility (MCC) tree with 10% burning using TreeAnotator (part of the BEAST package). The Bayesian skyline plot (BSKP) was built from the data to portray the genetic diversity of the AY.99.2 sublineage over time.

In parallel, we used the least square dating (LSD2) method implemented in IQ-TREE 2 to build a time tree using the tips information (days and months).

## Results

### GENOV Surveillance Program

**Figure 1:**
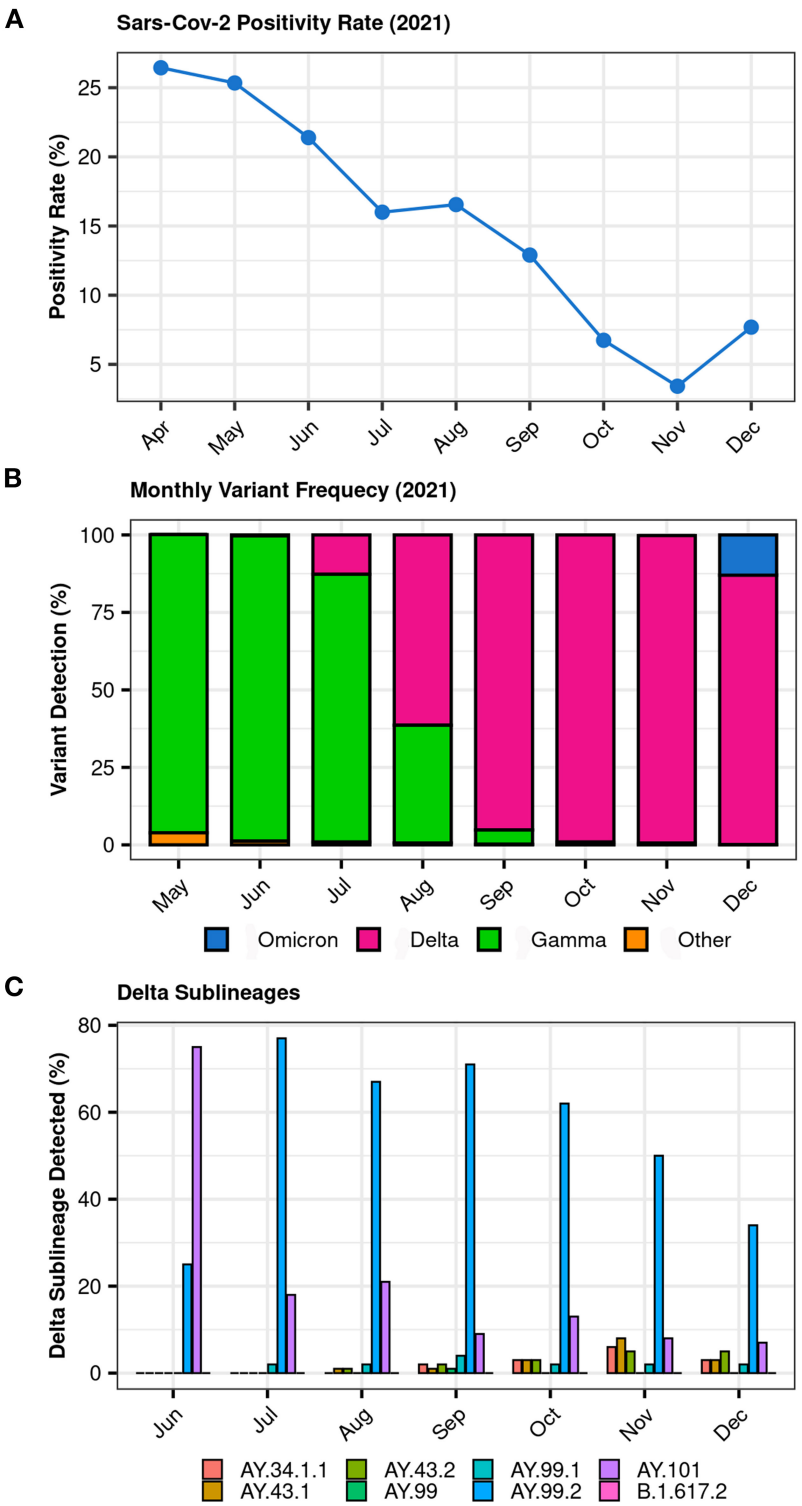
**(A)** SARS-CoV-2 RT-PCR positivity rate between April and December 2021 within Dasa samples; **(B)** Overall distribution of SARS-CoV-2 lineages between May and December 2021 (GENOV data); **(C)** Distribution of SARS-CoV-2 Delta sublineages between June and December 2021 (GENOV data).

The SARS-CoV-2 positivity rate within the Dasa samples between April and December 2021 decreased from 26.44 to 7.69%, with the lowest rate (3.42%) being observed in November (Figure 1A). The surveillance project GENOV started its activities in May 2021, when Gamma was still the dominant variant. From May to December 2021, the Dasa laboratories performed a total of 1,712,464 COVID-19 molecular tests (RT-PCR) from nearly all Brazilian regions. Of the positive samples, 12,054 were submitted to the complete genome sequencing and 9,181 had sufficient quality to be included in the further analysis. The analysis of these genomes reveals the replacement of the Gamma lineage by Delta, evident by August (Figure 1B). The Delta wave rose from late July (12.7%) peaking in November 2021 (≈99%) and starting to decrease by December 2021 (86.9%) as shown in Figure 1B. Among the Delta sublineages, the AY.99.2 was the most prevalent, with a rapid increase from 26% in June to 78% in July (Figure 1C).

### Global AY.99.2

Supporting the evidence that AY.99.2 emerged in Brazil, the first SARS-CoV-2 genomes from this lineage available in the GISAID database are from samples collected in April in the northern state of Ceará. In May, AY.99.2 isolates were detected also in the states of Rio Grande do Norte, São Paulo, and Minas Gerais. Besides Brazil, other countries detected this lineage only in July 2021, including South American neighboring countries.

**Figure 2:**
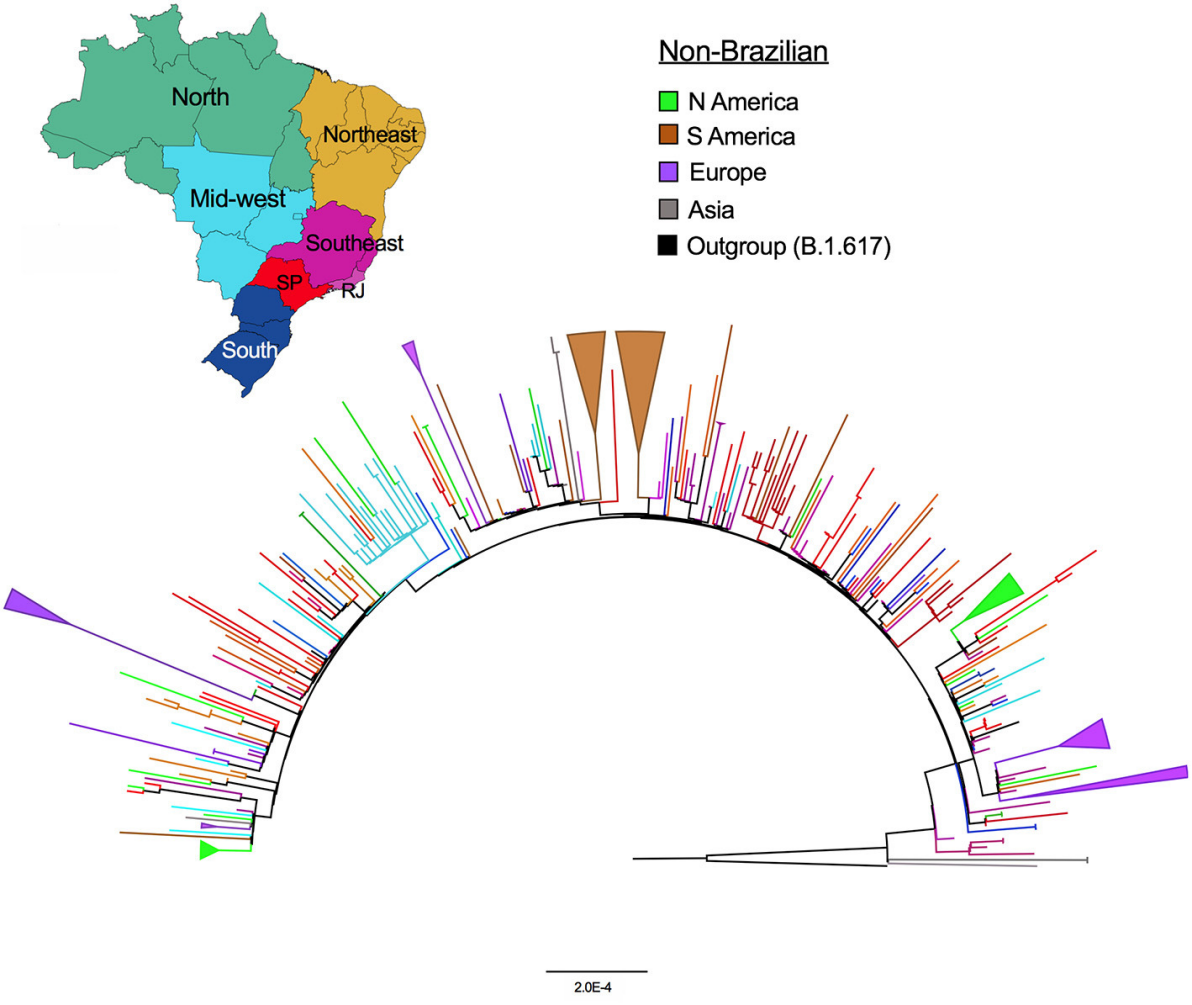
Maximum likelihood phylogenetic tree of AY.99.2 global sequences. The branches were colored according to the legend. The Brazilian sequences are colored by region as depicted on the map, note SP and RJ (red and light purple branches). Colored collapsed branches highlight the well-supported clades (>95% bootstrap) samples from Europe—purple; South America—brown; and North America—light green.

The global Delta AY.99.2 ML tree depicted in Figure 2 shows that while Brazilian sequences are more dispersed in the tree, non-Brazilian viruses tend to form small clusters, indicative of the independent origins of each one. The tree also reveals clusters of viruses sampled in North and South America and European countries (collapsed branches in light green, brown and purple), suggestive of separate introduction events. In Brazil, some small clusters are seen in Rio de Janeiro and São Paulo, which appear to be the main sources of the lineage dispersal. Distrito Federal, which also had a substantial number of sequences available, presented one large cluster (light blue) and a few sequences scattered along the tree.

**Figure 3:**
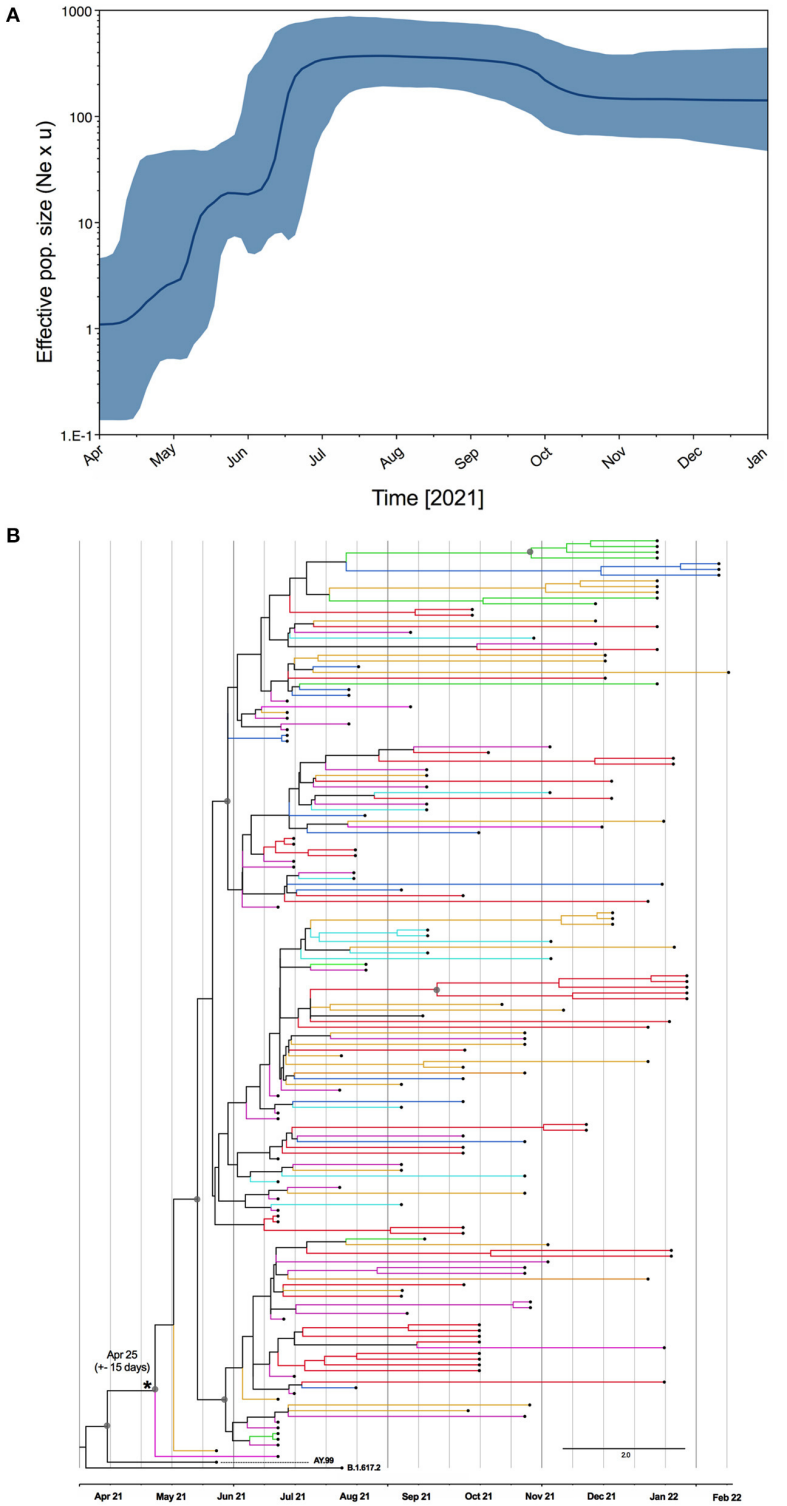
**(A)** Bayesian skyline reconstruction of AY.99.2 lineage of SARS-CoV-2 in Brazil. The Y-axis represents relative genetic diversity estimated through the effective population size (Ne) and the generation time (u) the thick solid line represents the median value of the estimates, and the gray area means the 95% HPD. **(B)** Maximum clade credibility tree reconstruction for Brazilian Delta AY.99.2. The branches are colored by region of sampling as depicted in the map in Figure 2. Gray circles highlight the nodes with posterior probability >0.7.

Since all evidence point to the Brazilian origin of this lineage, we estimated the time of the most recent common ancestor (tMRCA) for AY.99.2 in Brazil. Estimates were obtained using Bayesian inferences with a relaxed molecular clock in Beast, where we also deduced the bayesian skyline plot that explored the dynamics of it along with 2021, period where this lineage was largely spread in BR. The tMRCA was also estimated using maximum likelihood approach in iqtree2. Both methods indicated the origin of AY.99.2 at some point between April to middle May, 2021. According to the time-scaled maximum clade credibility tree (Figure 3B), the AY.99.2 emerged in late April (median 25 April +- 10 days), thus right after the introduction of the parent lineage B.1.617.2 in the country.

The Bayesian skyline plot (Figure 3A) illustrates an increase in the number of infections by the AY.99.2 lineage experienced between May and July, when this lineage was also exported to other countries and reach the highest variability. After July, a stasis in genetic diversity is seen, corresponding to a decrease in the number of covid-19 notifications in BR.

## Discussion

The SARS-CoV-2 positivity rate described by Dasa records reflects the pandemic in Brazil. Brazilian national data shows that in contrast to countries like England (15) and USA (16), the Delta introduction did not cause an increase in the number of cases or deaths in the country (17). A large number of cases in Europe and United States was attributed to the high infectiousness of Delta and the relaxing of restrictions policies, but thanks to the increased vaccination rate, the number of hospitalizations and deaths did not accompany the number of cases. Although Brazil was also under a vaccination campaign at that time, the rising of the vaccination rates was slow, and <20% of the population received at least one dose at the time of the Delta introduction to the country. Thus, a combination of factors may have contributed to the discrepancy observed among countries such as; differences in the transmission rates of the lineages, immune status of the population, demographic particularities and the non-pharmaceutical intervention policies then in place. Right before the Delta introduction, Brazil suffered with the severity of the Gamma VOC and its sublineages that caused a substantial number of infections and deaths all over the country. In fact, Brazil reached the peak in the number of deaths in late March to April 2021, when the astonishing record of 3k deaths in a single day was sadly recorded (18). It is possible that even Delta being more contagious than Gamma (19) the partial immunity of the population due to the recent massive Gamma infection (and in less proportion, due to vaccination) was determinant to limit the Delta expansion in this country.

### Delta Lineages-The Emergence and Spread

Different than other VOCs like Gamma or Omicron, Delta variant does not present the long branch signature, but instead it is characterized by a step-wise evolutionary process leading to the emergence of three clades and more than 240 sublineages in <1-year interval (20). Among the new sublineages, 7 are believed to have originated in Brazil.

After its first detection in Brazil in April 2021, the parental B.1.617.2 never became as prevalent in the country as its derived sublineages AY.99.2 and AY.101. By late August 2021, the AY.99.2 reached 60% prevalence among all SARS-CoV-2 lineages detected in the country (8).

The Delta “third wave” caused a sharp and rapid increase in the number of new cases worldwide. Even with efficient genomic surveillance programs, the number of SARS-CoV-2 genomes obtained during the “Delta wave” only represents a glimpse of the whole circulating genetic diversity. Despite this limitation, our analyses indicated that AY.99.2 emerged right after Delta arose in the country, and rapidly spread over the world. The data also suggest that the AY.99.2 was exported from Brazil (mainly from Sáo Paulo and Rio de Janeiro) to other countries multiple times, as indicated by the well-supported collapsed branches in the ML tree. At least 4 introductions of this lineage to European countries are observed; from Rio de Janeiro (RJ) to France, RJ to Portugal, São Paulo to Spain/Italy, and another cluster with an undefined source in Portugal. AY.99.2 genomes from South American neighbor countries as Argentina and Chile were more related to São Paulo and Rio de Janeiro lineages, respectively. North America sequences formed small clusters or did not cluster at all, and were more related to Distrito Federal and other sources suggesting several unrelated introductions in the USA states. Some small clusters of Sáo Paulo and Distrito Federal are also seen, but a within-country phylogeographic inference is compromised by the insufficient phylogenetic signal present in SARS-CoV-2, in particular in this situation where an explosion in the number of cases happened in such a small time-interval (21).

Independent data sources were consistent in depicting the rapid spread of Delta sublineages in Brazil. Particularly, the locally-emerged lineages AY.101 and AY.99.2 effectively surpassed the parental B.1.617.2. The AY.99.2 differs from its sister lineages AY.99 and AY.99.1 by two mutations (nuc: 4927C/T and ORF1a: T 4087 I). The non-synonymous homoplastic mutation in ORF1a is present in several VOC and non-VOC lineages and was not recognized as a “mutation of concern” according to the outbreak.info. Likewise, the mutational fitness estimated for AY.99.2 is 0.827 (22, 23). Therefore, no particular molecular characteristic that could result in better transmission fitness was described for these variants in comparison to the parental lineage that would explain the observed scenario. It is likely that the dominance of AY.99.2 in Brazil may have resulted from a chance “founder event,” where this lineage emerged and was established in a partially susceptible population and dominated the transmission network regardless of its fitness. Therefore, with few exceptions, AY.99.2 did not reach >1% prevalence in other countries (20).

The significance of rapid identification of a new variant of concern is unquestionable and the role of genomic surveillance to monitor the emergence and spread of better-fit VOCs has been proven crucial. However, as the epidemiological scenario evolves, we witness shifts in SARS-CoV-2 lineage composition in different geographic regions with distinct impacts. Our results support that the combination of a diverse array of data sources such as epidemiological and genomic data is the best way to monitor the impacts of spatial and temporal circulation of novel lineages of SARS-CoV-2.

## Data Availability Statement

The data presented in this study are deposited in GISAID (gisaid.org) and GenBank, under IDs #ON574629 - ON583798.

## Ethics Statement

The studies involving human participants were reviewed and approved by Ethical approval—CAAE 45540421.0.0000.5455. The patients/participants provided their written informed consent to participate in this study.

## Author Contributions

CR and CO were responsible for data analysis, interpretation of results, and wrote the manuscript. LS and CO were responsible for data acquisition and analysis and interpretation of data. JL was responsible for the study concept and design, data acquisition, and critical revision of the manuscript. All authors contributed to the article and approved the submitted version.

## Funding

This study was partially funded by KFW DEG Bank, grant G0512.

## Conflict of Interest

The authors declare that the research was conducted in the absence of any commercial or financial relationships that could be construed as a potential conflict of interest.

## Publisher's Note

All claims expressed in this article are solely those of the authors and do not necessarily represent those of their affiliated organizations, or those of the publisher, the editors and the reviewers. Any product that may be evaluated in this article, or claim that may be made by its manufacturer, is not guaranteed or endorsed by the publisher.
